# In Silico Models for Anti-COVID-19 Drug Discovery: A Systematic Review

**DOI:** 10.1155/2023/4562974

**Published:** 2023-06-15

**Authors:** Okello Harrison Onyango

**Affiliations:** Department of Biological Sciences, Molecular Biology, Computational Biology and Bioinformatics Section, School of Natural and Applied Sciences, Masinde Muliro University of Science and Technology, P.O. BOX 190, 50100 Kakamega, Kenya

## Abstract

The coronavirus disease 2019 (COVID-19) is a severe worldwide pandemic. Due to the emergence of various SARS-CoV-2 variants and the presence of only one Food and Drug Administration (FDA) approved anti-COVID-19 drug (remdesivir), the disease remains a mindboggling global public health problem. Developing anti-COVID-19 drug candidates that are effective against SARS-CoV-2 and its various variants is a pressing need that should be satisfied. This systematic review assesses the existing literature that used in silico models during the discovery procedure of anti-COVID-19 drugs. Cochrane Library, Science Direct, Google Scholar, and PubMed were used to conduct a literature search to find the relevant articles utilizing the search terms “In silico model,” “COVID-19,” “Anti-COVID-19 drug,” “Drug discovery,” “Computational drug designing,” and “Computer-aided drug design.” Studies published in English between 2019 and December 2022 were included in the systematic review. From the 1120 articles retrieved from the databases and reference lists, only 33 were included in the review after the removal of duplicates, screening, and eligibility assessment. Most of the articles are studies that use SARS-CoV-2 proteins as drug targets. Both ligand-based and structure-based methods were utilized to obtain lead anti-COVID-19 drug candidates. Sixteen articles also assessed absorption, distribution, metabolism, excretion, toxicity (ADMET), and drug-likeness properties. Confirmation of the inhibitory ability of the candidate leads by *in vivo* or *in vitro* assays was reported in only five articles. Virtual screening, molecular docking (MD), and molecular dynamics simulation (MDS) emerged as the most commonly utilized in silico models for anti-COVID-19 drug discovery.

## 1. Introduction

COVID-19 is one of the most significant infectious illnesses in the world. It affects hundreds of millions annually and is the main factor behind socioeconomic loss in developing nations. As of 21st March 2023, the World Health Organization (WHO) reported approximately 761,071,826 COVID-19 cases and 6,879,677 confirmed deaths [1]. The emergence of SARS-CoV-2 variants and the availability of only one FDA-approved anti-COVID-19 drug are the main issues in controlling and treating COVID-19. Therefore, the need to find effective anti-COVID-19 drug candidates is crucial. However, finding and developing new drugs takes time and money [2]. According to the 2016 Tufts Center for the Study of Medicine Development research, developing a new drug typically takes more than ten years and costs more than $2.6 billion [3]. Executing an ideal drug discovery and development method is one of the main issues facing the pharmaceutical research community [4]. In silico drug design and development represents a technique to expedite drug discovery and development procedures efficiently as one of the main aims.

In silico drug design and discovery is a rigorous procedure of finding novel drugs based on understanding a biological target. It is essential to create tiny molecules for complementary drugs in charge and form the biomolecular targets they interact with [5]. Discovering small compounds that preferentially bind to the biological target with the highest binding affinity is crucial. Finding and developing novel drugs faces new challenges and opportunities due to recent advances in bioinformatics and other omics approaches like genomics and proteomics. Several disciplines such as computer science, biological sciences, and information technology or informatics have greatly benefited from the fusion of these technological advances. Protein networks and other fast-developing information on drug-target interactions (DTI), gene expression, and other topics are becoming more widely available and standardized [6].

Through the use of numerous readily accessible databases of chemical compounds and proteins like protein data bank (PDB) where SARS-CoV-2 target proteins, such as main protease (M^pro^), spike protein, and RNA-dependent RNA polymerase (RdRp), can be retrieved, in silico or computational-based methods can speed up the development process for anti-COVID-19 drugs. During the computer-aided drug discovery process, the costs are often insignificant because humans are rarely in danger, expenditures are negligible, and biosafety facilities are unnecessary [5]. However, despite discovering new drugs through in silico means, several usually fail during clinical trials due to toxicity and poor pharmacokinetics features. These pharmacokinetics characteristics, including ADMET, are crucial for discovering and developing new medicines [5]. This is evident in the studies by Adel et al. [7] and Shabaan et al. [8] that performed ADMET properties analysis of potential anti-COVID-19 compounds. Therefore, subjecting newly discovered drug candidates to ADMET or pharmacokinetics properties analysis and prediction can assist in eliminating molecules with unfavorable drug ability characteristics. In silico tools like SwissADME can be utilized for ADMET or pharmacokinetics properties analysis and forecast of drug candidates [2, 7, 8]. Similarly, molecular modeling can be applied to ADMET or the candidate compounds' pharmacodynamics (toxicity and drug action) characteristics [9].

Computer-aided drug design and discovery have been accomplished via structure- and ligand-based drug development [10]. The structure-based drug design is called direct drug design [5]. It provides an avenue to create new molecular entities interacting with specific biological targets using a model of the said targets [11]. Structure-based drug design and development require an in-depth understanding of the biological target's three-dimensional structure. NMR spectroscopy and X-ray crystallography are techniques used to attain the three-dimensional structures of biological targets [12]. These three-dimensional structures are often handy when computational approaches like 3D-QSAR involving force field calculations are performed based on molecular superimposition or protein crystallography. With the help of interactive visuals, a medicinal chemist's intuition, and several automated computational techniques, candidate medications that are anticipated to bind to a particular biological target with high selectivity and affinity can be created [10]. On the other hand, ligand-based drug design and discovery, also called indirect drug design, requires a profound comprehension of other compounds (ligands) that attach to the desired biological target [5]. In some instances, such molecules can create one reference ligand that functions as a pharmacophore model, establishing the minimum requirements for a molecule to bind to a target, as evident in Onyango et al. [2]. This systematic review analyzed research publications employing in silico techniques to find new anti-COVID-19 drugs, summarized, and presented that information, which is crucial for further discovery and development of effective anti-COVID-19 medications.

## 2. Materials and Methods

### 2.1. Study Design

This systematic review evaluated the computational in silico models used to discover new anti-COVID-19 drugs.

### 2.2. Literature Searches

The following electronic databases were searched for research studies published between January 2019 and the end of December 2022: PubMed, Google Scholar, Cochrane Library, and Science Direct, according to the Preferred Reporting Items for Systematic Reviews and Meta-analysis (PRISMA). The Boolean operators “OR” and “AND” were also used to help in the literature search by combining the following keywords and terms “Drug Discovery,” “Anti-COVID-19,” “In Silico Models,” “Computer-Aided Drug Design,” and “Drug Development.” Since language restrictions do not affect or alter the results of systematic reviews, all searches were limited to studies published in English. Additional articles were searched by thoroughly examining the reference lists of research publications retrieved from the electronic databases. Duplicate research publications were documented and omitted from the review.

### 2.3. Eligibility Criteria

All research publications that employed different in silico models for discovering novel anti-COVID-19 drugs published in English from 2019 to December 31, 2022, were included in the study. Studies were not fully accessible by virtue of being behind a paywall, publications on diseases other than COVID-19, and duplicate articles were excluded.

### 2.4. Study Selection Process

The searched studies from all databases were randomly downloaded to reduce the chances of bias. All downloaded publications were individually reviewed to determine their eligibility. The titles and authors of each research study were examined, and duplicates were removed. All downloaded publications' titles and abstracts were then screened for potential relevance. The full-text review was performed on the studies for which there was uncertainty about significance. Publications that failed to meet the inclusion criteria were excluded.

### 2.5. Data Extraction and Synthesis

The data were manually extracted from the research publications and recorded in a table. The following data were extracted from each of the research articles included in the review: the title of the article, the reference (names of the authors and years of publication), in silico methods used (2- or 3-D Quantitative Structure-Activity Relationship: QSAR, pharmacophore modeling, MD, homology modeling, and others), software packages and web-based databases and servers utilized, biological targets, lead or hit compounds, and experimental techniques (*in vivo* or *in vitro* assays) where applicable. Thematic analysis was used to synthesize the data, and similar information was grouped into columns, as displayed in Table 1.

## 3. Results

### 3.1. Study Selection

**Figure 1:**
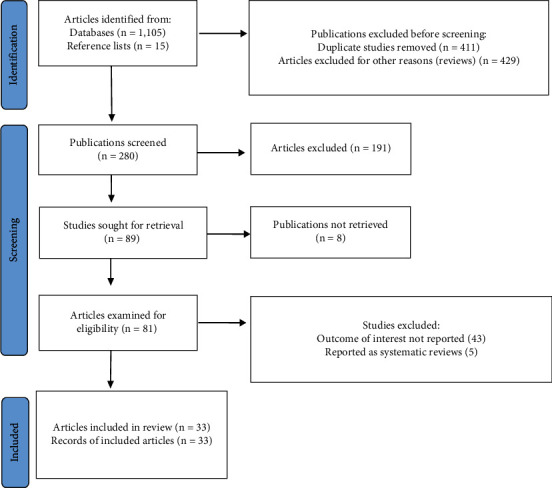
PRISMA chart displaying the different phases of the systematic literature review. 1,120 publications were retrieved from electronic databases and reference lists. 840 articles were removed because they were duplicates and reviews. 191 were excluded because of nonrelevance after the screening. Eight of 81 articles could not be recovered, and 48 were excluded because they failed to report the outcome of interest. Therefore, 33 studies were included in the review.

A comprehensive search of the electronic databases yielded 1,105 possibly associated research publications based on the keywords and search terms described previously, published between 2019 and 2022 (Figure 1). A thorough review of the reference lists of some of these electronically retrieved research articles provided 15 additional publications linked to the topic of interest. Therefore, 1,120 research articles were obtained from the initial phase of the literature search. The titles of these 1,120 articles were checked to identify duplicates and studies that were not original research, for instance, reviews. From these initial publications, 840 articles were excluded for the following reasons: duplicates (411) and review publications (429). From the remaining 280 articles, 191 were excluded after a review of their titles and abstracts confirmed nonrelevance to this review. Therefore, 89 full-text publications were sought for retrieval. However, only 81 full-text publications could be retrieved. The full texts of 8 publications were inaccessible. In this regard, 81 articles were examined for eligibility based on the preset criteria, further excluding 48 studies primarily due to their failure to report the outcome of interest. Eventually, 33 publications met the eligibility criteria and were included in the final review (Figure 1 and Table 1).

### 3.2. Study Characteristics

Table 1 summarizes the information collected from the 33 articles included in this current systematic review. The data were categorized into different themes: title of the article, reference (authors and year of publication), in silico methods/software/databases used, drug target, lead candidates, and experimental technique. Table 1 shows that only one author published one article [37]. The other three articles were published by two authors [23, 32, 41]. More than two authors published all the remaining twenty-nine studies. All articles had different titles directly associated with the topic of interest. They were published during different periods within the 2019–2022 timeframe. 9.09% of the articles were published in 2020. 57.58% of the reports were published in 2021. 33.33% of the studies were published in 2022.

The published in silico models were mainly applied for identifying prospective anti-COVID-19 drugs using SARS-CoV-2 proteins and some human proteases as targets. Seven studies undertook pharmacophore modeling, chemical synthesis of ligands, ligand database creation, or homology modeling as preliminary steps of anti-COVID-19 drug discovery. 16 publications performed virtual screening to discover compounds with inhibitory effects on SARS-CoV-2 target proteins. MD emerged as a crucial step in finding anti-COVID-19 drugs. Twenty-six articles employed MD to test the binding affinities of their lead compounds to SARS-CoV-2 target proteins. Another in silico process that was common was molecular dynamics simulation. Twenty-three studies applied the method to ascertain the stability of their ligand-target protein complexes. After MDS, 25 publications underscored the need for drug-likeness and physicochemical and pharmacokinetics assessment. Ten reports analyzed the drug-likeness of their lead compounds, while 15 articles assessed their drug candidates' physicochemical and pharmacokinetics properties. These two in silico procedures were performed to virtually confirm the lead compounds' drug ability.

**Figure 2:**
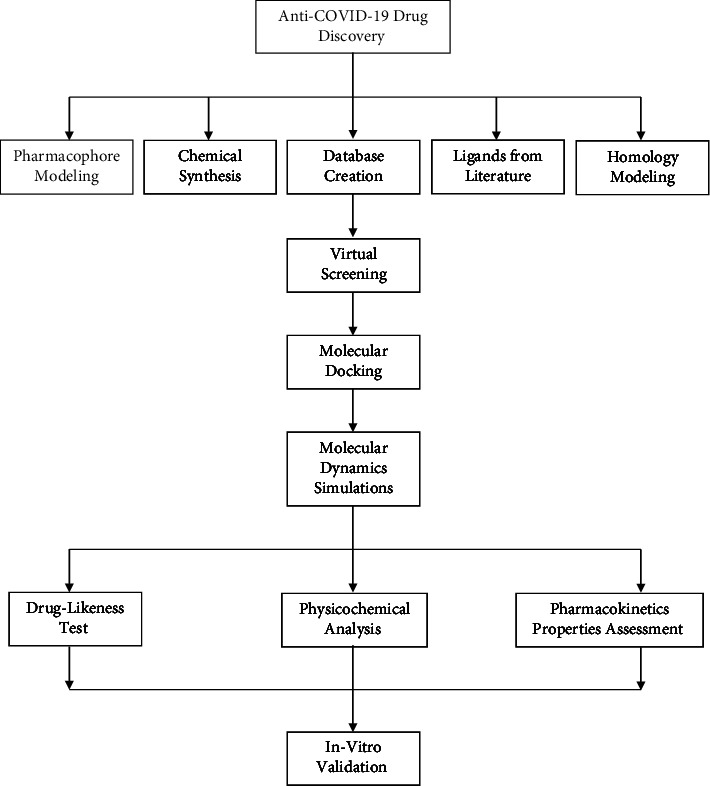
Flowchart diagram that summarizes the most commonly used in silico models for anti-COVID-19 drug discovery.

The lead candidates from each publication depended on the drug target and the databases used for virtual screening. Therefore, the lead candidates ranged from diazole, furan, and pyridine to gliquidone, glimepiride, and linagliptin (Table 1). However, the drug targets used were 9: RdRp (5 articles), spike protein (5 articles), main protease (25 publications), nsp16 (2 articles), nsp15 (1 article), nsp12 (1 study), PL^pro^ (3 studies), TMPRSS2 (2 publications), and ACE2 (5 articles). The most common drug target in the fight against COVID-19 is SARS-CoV-2 main protease. Even though human proteases or enzymes like ACE2 and TMPRSS2 are also used, SARS-CoV-2 proteins are preferred. Some studies (5) performed in silico approaches and *in vitro* validation of their lead candidates. Although the remaining 28 articles did not undertake *in vitro* validation of their drug candidates, they recommended additional clinical processes to ascertain the use of their lead compounds as anti-COVID-19 drugs.  Figure 2 is a flowchart diagram summarizing the most commonly used in silico models for anti-COVID-19 drug discovery.

### 3.3. Information from Individual Studies

This review discovered different in silico models used for anti-COVID-19 drug discovery. For instance, chemical synthesis/Cu(I)-catalyzed click 1,3-dipolar cycloaddition reaction, molecular docking using MOE 2019, and physicochemical properties and drug-likeness test using Molinspiration and Mol-Soft software are some of the in silico approaches and tools used for anti-COVID-19 drugs discovery [13]. In this study, Alzahrani et al. [13] used RNA-dependent RNA polymerase, spike protein S1, main protease (3CLpro), and 2′-O-methyltransferase (nsp16) as drug targets. The researchers discovered bis-(1,2,3-triazole-sulfa drug hybrids) carrying benzimidazole moiety (4b and 4c) as lead compounds against RNA-dependent RNA polymerase, 4c against SARS-CoV-2 spike protein, and 4b and 4c against SARS-CoV-2 3CLpro and nsp16. The study also performed *in vitro* validation of these lead compounds.

Other researchers who used different types of *in vitro* validation in their studies were Lin et al. [18] (SPR assay), Zhao et al. [19] (cell-based assays), Rabie [37] (*in vitro* anti-COVID-19 bioactivity of Taroxaz-104), and Qu et al. [44] (*In vitro* study). These four studies employed similar in silico models. For instance, all of them used molecular docking and molecular dynamics simulation. In addition to the two in silico techniques, Lin et al. [18] and Zhao et al. [19] utilized virtual screening to search libraries of small molecules to detect those compounds that can bind to specific drug targets of interest, including SARS-CoV-2 main protease [18] and SARS-CoV-2 papain-like protease (PLpro) and SARS-CoV-2 main protease [19]. Rabie [37] and Qu et al. [44] used RNA-dependent RNA polymerase (nCoV-RdRp) and SARS-CoV-2 M^pro^, respectively, as their drug targets. The studies discovered Yinqiao powder [18], YM155 [19], Taroxaz-104 [37], and repaglinide, canagliflozin, glipizide, gliquidone, glimepiride, and linagliptin [44] as their lead compounds.

SARS-CoV-2 M^pro^ was already mentioned as the most common drug target. Several researchers used it in their studies as the preferred drug target, including Ghosh et al. [14]; Kumari et al. [16]; Ibrahim et al. [20]; Ibrahim et al. [21]; Rivero Segura and Gomez-Verjan [23]; Ibrahim et al., [27]; Feng et al., [31]; Adem et al., [33]; Elkaeed et al., [34]; Ibrahim et al., [35]; Ahmad et al., [36]; Ibrahim et al., [39]; and Aleissa et al., [40]. All these researchers used SARS-CoV-2 M^pro^ as the sole drug target and discovered different lead compounds that could be used as anti-COVID-19 drugs. Those drug candidates include diazole, furan, and pyridine [14], doxorubicin and budesonide (pulmicort) [16], salvianolic acid A and curcumin [20], TMC-310911 and ritonavir [21], cichoriin [23], DB02388 and cobicistat [27], lopinavir, tenofovir disoproxil, fosamprenavir, ganciclovir, peramivir, zanamivir, and sofosbuvir [31], hesperidin, rutin, diosmin, and apiin [33], luteoside C, kahalalide E, and streptovaricin B [34], four bis [7, 36] dioxolo pyran-5-carboxamide derivatives [35], SCHEMBL 12616233, SCHEMBL 18616095, and SCHEMBL 20148701 [36], PubChem-129-716-607 and PubChem-885-071-27 [39], and HIT-1 and HIT-2 [40].

Even though all these researchers did not perform *in vitro* validation of the inhibitory ability of these lead compounds, they utilized relatively comparable in silico approaches to discover them. Gosh et al. [14] employed QSAR/SiRMS tools for anti-COVID-19 drug discovery. All other studies utilized molecular docking in addition to chemical-chemical and chemical-protein interactions using the STITCH database and randomization test using SWISSADME [16], molecular dynamics simulation, drug-likeness tests, and protein-protein interactions [20], molecular dynamics simulation [21, 27], virtual screening and pharmacokinetic assessment [23], virtual screening by MCCS [31], quantum mechanics and molecular dynamic simulations [33], molecular similarity detection using Discovery Studio software, molecular fingerprint detection using Discovery Studio software, toxicity studies using Discovery Studio 4.0, and molecular dynamics (MD) simulations using the GROningen machine [34], virtual screening of MolPort database, molecular dynamics (MD) simulations, and drug-likeness predictions [35, 39, 40], and structure-based virtual screening (SBVS) of ASINEX antiviral library, drug-likeness and lead likeness annotations, pharmacokinetics analysis, and molecular dynamics (MD) simulations [36].

Other researchers preferred using SARS-CoV-2 M^pro^ with other SARS-CoV-2 proteins or human proteases as their drug targets. For example, Ongtanasup et al. [22]; Xu et al. [25]; Shahabadi et al. [26]; and Wang et al. [28] used SARS-CoV-2 M^pro^ and ACE2 as their drug targets. Ongtanasup et al. [22] undertook MD, MDS, drug-likeness, and ADMET prediction and found *Myristica fragrans* compounds as suitable drug candidates against COVID-19. Xu et al. [25] utilized the same in silico techniques in addition to virtual screening and identified red wine, Chinese hawthorn, and blackberry as substances with anti-COVID-19 compounds. Shahabadi et al. [26] undertook only two in silico processes, MD and MDS to find cetilistat, abiraterone, di-iodo hydroxyquinoline, and bexarotene as anti-COVID-19 drug candidates. Among the four groups of scholars, Wang et al. [28] employed seven in silico models in their anti-COVID-19 drug discovery process. The authors used virtual screening, molecular interaction networks using Cytoscape, protein–protein interaction (PPI) network construction, gene ontology enrichment analysis, KEGG pathway analysis, molecular docking, and molecular dynamics (MD) simulation. They found compounds created using the HuaShi XuanFei Formula (HSXFF) as probable anti-COVID-19 drugs.

SARS-CoV-2 M^pro^ has also been used in combination with ACE2 and PL^pro^ [29], spike protein [30], and PL^pro^, RdRp, nsp15, and spike protein [45] as drug targets. With these targets, Shawan et al. [29] created a flavonoid library, performed virtual screening, MD, and MDS, and assessed the drug-likeness and ADMET profiles of its final lead compounds: luteolin and Abyssinian II. Quimque et al. [45] utilized the same in silico models. They discovered three fumiquinazoline alkaloids, scedapin C, quinadoline B, and norquinadoline A, the polyketide iso-chaetochromin, and the terpenoid 11a-de-hydroxy isoterreulactone A as potential drugs against COVID-19. Arunkumar et al. [30] performed molecular docking using AutoDock tools, MDS, and ADMET and density functional theory calculations to discover drug candidates, mainly k-carrageenan, laminarin, eckol, trifucol, and bD-galactose.

Human proteases such as TMPRSS2 [17, 24] and other SARS-CoV-2 proteins such as RdRp [32, 42], spike protein [43], nsp12 [41], SARS-CoV-2 spike receptor-binding domain (RBD) [38], and nsp16 [15] have also been used as the sole drug targets in specific studies. In those respective studies, Shi et al. [15] discovered C1 with CAS ID 1224032-33-0 and C2 with CAS ID 1224020-56-7 as nsp16 inhibitors after employing the following in-silico techniques: pharmacophore modeling using phase, pharmacophore-based virtual screening using phase, MD using glide, and MDS using Gromacs 2021. Alzain et al. [17] and Wang et al. [24] identified groups of drugs abbreviated as combine 1, 2, and 3 and lumacaftor and ergotamine, respectively, using similar in silico processes: homology modeling, high-throughput virtual screening, molecular docking, and MDS. Bharti and Shukla [32] and Baby et al. [42], who used the same drug target, discovered different anti-COVID-19 drug candidates. Bharti and Shukla [32] found ellipticine, ecteinascidin, homo harringtonine, dolastatin 10, halichondrin, and plicamycin after performing molecular docking, absorption, distribution, metabolism, and excretion (ADME) assessment, and drug-likeness test. On the other hand, Baby et al. [42] identified pitavastatin, ridogrel, and rosoxacin after executing only molecular docking and MDS.

Muhseen et al. [38] opted for MDS and structure-based virtual screening to obtain NPACT01552, NPACT01557, and NPACT00631 as probable inhibitors of the SARS-CoV-2 spike receptor-binding domain (RBD). Rao and Shetty [41] performed virtual screening, pharmacokinetic and pharmacodynamics properties examination, molecular docking, and MDS and discovered 12,28-oxa-8-hydroxymanzamine A as a potential inhibitor of nsp12. In the last study, Pandey et al. [43] utilized the same in silico models employed by several other researchers: molecular docking, MDS, and ADME analysis. The authors found fisetin, quercetin, and kaempferol as lead compounds against COVID-19.

## 4. Discussion

### 4.1. Drug Targets

Several drug targets were identified and validated using in silico approaches. In the fight against COVID-19, this study's findings align with information in existing literature on the drug targets being SARS-CoV-2 proteins. The most common is SARS-CoV-2 M^pro^, also referred to as 3-chymotrypsin-like proteases (3CLpro). It is a highly conserved cysteine hydrolase in *β*-coronaviruses with an essential function *in viral* replication. It is a key target for treating and preventing infectious diseases caused by coronavirus, including COVID-19 [46]. Other SARS-CoV-2 proteins utilized as drug targets include SARS-CoV-2 ribonucleic acid (RNA)-dependent RNA polymerase (RdRp), SARS-CoV-2 spike protein, nsp16, SARS-CoV-2 PL^pro^, and nsp12. SARS-CoV-2 RdRp is a viral enzyme responsible for viral RNA replication in host cells [47]. Zhu et al. [47] explain that SARS-CoV-2 RdRp has no host cell homologs. Therefore, its inhibitors can be created with improved potency and fewer off-target impacts on human host proteins and thus are more effective and safer therapeutics for treating COVID-19. SARS-CoV-2 RdRp has a catalytic subunit called nonstructural protein 12 (nsp12). Hillen et al. [48] outline that with the help of conserved residues, the active-site cleft of nsp12 attaches to the first turn of RNA and regulates RdRp action. Therefore, nsp12 can also be used as a drug target because it mediates the SARS-CoV-2 RdRp function.

Among all human coronaviruses, this study's findings conform with information in existing literature that the SARS-CoV-2 spike protein is highly conserved and takes part in the recognition of the receptor, attachment of the virus, and viral entry into host cells. Due to its vital role, it embodies one of the most significant targets for COVID-19 therapeutic and vaccine research [49]. Even though nsp16 and PL^pro^ are drug targets, they are rarely used in anti-COVID-19 drug development. Between the two, the 2′-O-methyltransferase nonstructural protein 16 (Nsp16) is crucial for immunological evasion. Nsp16 does this by imitating CMTr1, its human ortholog, which methylates mRNA to improve translation efficacy and differentiate itself from each other [50]. One of the two SARS-CoV-2 protease antivirals that could potentially target is a papain-like protease (PL^pro^). Because it is crucial for viral polyproteins' cleavage and maturation, the construction of the replicase-transcriptase complex, and interference with host defenses, PL^pro^ is also a desirable target [51]. The last two drug targets are human proteases called TMPRSS2 and ACE2. SARS-CoV-2 requires the serine protease TMPRSS2 for S protein priming and the SARS-CoV receptor ACE2 for entry [52]. Therefore, TMPRSS2 and ACE2 inhibitors can restrict entry and be a therapy option.

### 4.2. Lead Identification Process

Most studies in this review, as evident in existing literature as well, employed in silico modeling for ligand-based and structure-based design for probable anti-COVID-19 drug candidates using known targets already described. The lead identification process encompassed all necessary anti-COVID-19 drug discovery processes. As shown in Figure 2, the first in silico processes that most studies employ include pharmacophore modeling, chemical synthesis, database creation, homology modeling, and literature search. Pharmacophore modeling involves using several ligands to create a model with common pharmacophore features. The model, popular as a pharmacophore, is a collection of electronic and steric characteristics that ascertain optimal supramolecular interactions during virtual screening on large-scale compound databases [53]. Before virtual screening of the databases, other researchers opt to perform homology modeling or chemical synthesis of their ligands of interest. Homology modeling involves predicting the 3D structure of a ligand using its amino acid sequence. At the same time, chemical synthesis refers to using one or more chemical reactions to convert a starting material into a desired ligand or compound. Since several antiviral compounds are well-known and their structures already elucidated, some scholars prefer performing literature searches and creating a database of such molecules that they use for further drug discovery processes.

The next three in silico processes usually include virtual screening, molecular docking, and molecular dynamics simulation. Virtual screening is an in-silico technique used in drug development to find the structures most probable to bind to a therapeutic target, often a protein, enzyme, or receptor. The selected hit molecules obtained by virtual screening are subject to molecular docking, which estimates the binding energy and interaction affinity involved in the interaction between a receptor and a ligand [2]. The ligand-receptor complexes with the best binding affinity undergo molecular dynamics simulation, enhancing the comprehension of a system's dynamic performance. It measures the stability of a complex. Determining stable complexes is a step further toward developing a drug. However, drug-likeness and ADMET properties analysis are essential to determine the desirability of a lead compound as an anti-COVID-19 drug. Evaluating the absorption, distribution, metabolism, excretion, and toxicity (ADMET) properties of lead compounds is one of the significant criteria before developing a compound into a drug because they shed some light on the molecules' solubility, GIT absorption, and bioavailability profiles. These processes underscore the basic in silico models that several researchers employ. However, not all researchers adhere to the methodology described previously. The procedure they adopt depends on numerous factors such as preference, objectives, databases used, and others. Combining two or more of these techniques must be employed during anti-COVID-19 drug discovery.

## 5. Conclusion

A practical approach for identifying possible anti-COVID-19 drug targets and probable lead compounds during the drug discovery phase is in silico modeling. Clarifying their mechanisms of action and possible medical usefulness is another benefit of using in silico models. An existing literature agrees that using in silico and other computation techniques to investigate possible medications is a secure, affordable, and efficient way to find, create, or repurpose potential remedies. Even though medical and nonclinical validation employing some *in vivo* and *in vitro* assays is still required to further confirm the antiviral activity of these possible candidate molecules, discovering those particular lead compounds using in silico means is a step in the right direction when drug design, development, and discovery are concerned. This study in unison with existing literature confirms that the in silico methods that use several drug targets have the best chance of succeeding because of the broad scope of potential lead candidates. Additionally, the in silico methods should be used concurrently to forecast ADMET and drug-like features of the candidate compounds.

## Figures and Tables

**Table 1:** Description of the studies included in the review, including methods or software applied for in silico discovery of anti-COVID-19 drugs.

Title	Reference	Method/software or databases applied	Drug target	Lead candidate	Experimental technique
Anti-COVID-19 activity of some benzofused 1,2,3-triazolesulfonamide hybrids using *in silico* and *in vitro* analyses	Alzahrani et al. [13]	Chemical synthesis/Cu(I)-catalyzed click 1,3-dipolar cycloaddition reaction	RNA-dependent RNA polymerase	Bis-(1,2,3-triazole-sulfadrug hybrids) carrying benzimidazole moiety (4b and 4c) against RNA-dependent RNA polymerase	*In vitro* antiviral activity
		Molecular docking/MOE 2019	Spike protein S1 main protease (3CLpro)	4c against SARS-CoV-2 spike protein	
		Physicochemical properties and drug-likeness test/molinspiration and Mol-Soft software	2′-O-methyltransferase (nsp16)	4b and 4c against SARS-CoV-2 3CLpro and nsp16	
Chemical-informatics approach to COVID-19 drug discovery: the exploration of important fragments and data mining based prediction of some hits from natural origins as main protease (Mpro) inhibitors	Ghosh et al. [14]	QSAR/SiRMS tools	SARS-CoV-2 M^pro^	Diazole, furan, and pyridine	None

Computational investigation of potent inhibitors against SARS-CoV-2 2′-O-methyltransferase (nsp16): structure-based pharmacophore modeling, molecular docking, molecular dynamics simulations, and binding free energy calculations	Shi et al. [15]	Pharmacophore modeling/phase	SARSCoV-2 2′-O-methyltransferase (nsp16)	C1 with CAS ID 1224032-33-0 and C2 with CAS ID 1224020-56-7	None
		Pharmacophore-based virtual screening/phase			
		Molecular docking/glide			
		Molecular dynamics simulation/Gromacs 2021			

Discovery of new drug indications for COVID-19: A drug repurposing approach	Kumari et al. [16]	Chemical-chemical and chemical-protein interaction/STITCH database	SARS-CoV-2 M^pro^	Doxorubicin and buedesonide (pulmicort)	None
		Randomization test/SWISSADME			
		Molecular docking/Autodock 4 tool			

Discovery of novel TMPRSS2 inhibitors for COVID-19 using in silico fragment-based drug design, molecular docking, molecular dynamics, and quantum mechanics studies	Alzain et al., [17]	Homology modeling using Schrodinger	TMPRSS2	Combine 1, 2, and 3	None
		Program			
		High-throughput virtual screening			
		Molecular docking			
		Molecular dynamics simulation			

Exploring the treatment of COVID-19 with Yinqiao powder based on network pharmacology	Lin et al., [18]	Virtual screening	SARS-CoV-2	Yinqiao powder	SPR assay
		Protein-protein interaction network construction			
		Molecular docking			

High-throughput screening identifies established drugs as SARS-CoV-2 PLpro inhibitors	Zhao et al., [19]	Virtual screening	SARS-CoV-2 papain-like protease (PLpro)	YM155	Cell-based assays
			SARS-CoV-2 main protease		

In silico drug discovery of major metabolites from spices as SARS-CoV-2 main protease inhibitors	Ibrahim et al., [20]	Molecular docking	SARS-CoV-2 main protease	Salvianolic acid A and curcumin	None
		Molecular dynamics simulation			
		Drug-likeness			
		Protein-protein interaction			

In silico evaluation of prospective anti-COVID-19 drug candidates as potential SARS-CoV-2 main protease inhibitors	Ibrahim et al., [21]	Molecular docking	SARS-CoV-2 main protease	TMC-310911 and ritonavir	None
		Molecular dynamics simulation			

In silico investigation of ACE2 and the main protease of SARS-CoV-2 with phytochemicals from Myristica fragrans (Houtt.) for the discovery of a novel COVID-19 drug	Ongtanasup et al., [22]	Molecular docking	ACE2 and the main protease of SARS-CoV-2	*Myristica fragrans* compounds	None
		Molecular dynamics simulation			
		Drug-likeness and absorption, distribution, metabolism, excretion, and toxicity (ADMET) prediction			

In silico screening of natural products isolated from Mexican herbal medicines against COVID-19	Rivero-Segura and Gomez-Verjan [23]	Virtual screening	SARS-CoV-2 proteins	Cichoriin	None
		Molecular docking			
		Pharmacokinetic assessment			

In silico screening of novel TMPRSS2 inhibitors for treatment of COVID-19	Wang et al., [24]	Homology modeling and virtual screening	TMPRSS2	Lumacaftor and ergotamine	None
		Molecular dynamics simulation			

In silico screening of potential anti-COVID-19 bioactive natural constituents from food sources by molecular docking	Xu et al., [25]	Virtual screening	SARS-CoV-2 CL^pro^	Red wine, Chinese hawthorn, and blackberry	None
		Molecular docking	Humans ACE2		
		ADME analysis			
		Drug likeness			

Inhibitory activity of FDA-approved drugs cetilistat, abiraterone, diiodohydroxyquinoline, bexarotene, remdesivir, and hydroxychloroquine on COVID-19 main protease and human ACE2 receptor: A comparative in silico approach	Shahabadi et al., [26]	Molecular docking	SARS-CoV-2 main protease	Cetilistat, abiraterone, di-iodo hydroxyquinoline, and bexarotene	None
		Molecular dynamics simulation	ACE2		

In-silico drug repurposing and molecular dynamics puzzled out potential SARS-CoV-2 main protease inhibitors	Ibrahim et al., [27]	Molecular docking	SARS-CoV-2 main protease	DB02388 and cobicistat	None
		Molecular dynamics simulation			

Investigating the active compounds and mechanism of HuaShi XuanFei formula for prevention and treatment of COVID-19 based on network pharmacology and molecular docking analysis	Wang et al., [28]	Virtual screening	3C-like (3CL) protease hydrolase and angiotensin-converting enzyme 2 (ACE2)	HuaShi XuanFei	None
		Molecular interaction networks using Cytoscape		Formula (HSXFF)	
		Protein–protein interaction (PPI) network construction			
		Gene ontology enrichment analysis and KEGG pathway analysis			
		Molecular docking			
		Molecular dynamic (MD) simulation			

Luteolin and abyssinone II as potential inhibitors of SARS-CoV-2: an in silico molecular modeling approach in battling the COVID-19 outbreak	Shawan et al., [29]	Creation of flavonoids library	ACE2 of human host and Mpro/3CLpro and PLpro of SARS-CoV-2	Luteolin and abyssinone II	None
		Drug likeness/pharmacophore and ADMET profile analysis			
		Virtual screening and molecular docking			
		Molecular dynamics simulation			
		ADMET profile analysis			

Marine algal antagonists targeting 3CL protease and spike glycoprotein of SARS-CoV-2: a computational approach for anti-COVID-19 drug discovery	Arunkumar et al., [30]	Molecular docking tools (AutoDockTools)	3CL protease and spike glycoprotein of SARS-CoV-2	k-Carrageenan, laminarin, eckol, trifucol, and b-D-galactose	None
		Molecular dynamic simulation, ADMET, and density functional theory calculations			

MCCS: a novel recognition pattern-based method for fast-track discovery of anti-SARS-CoV-2 drugs	Feng et al., [31]	Virtual screening by MCCS	3CL^Pro^ in SARS-CoV-2	Lopinavir, tenofovir disoproxil, fosamprenavir, and ganciclovir	None
				Peramivir and zanamivir	
				Sofosbuvir	

Molecules against Covid-19: an in silico approach for drug development	Bharti and Shukla [32]	Molecular docking	SARS-CoV-2 ribonucleic acid (RNA)-dependent RNA polymerase (RdRp)	Ellipticine, ecteinascidin, homo harringtonine, dolastatin 10, halichondrin, and plicamycin	None
		Absorption, distribution, metabolism, and excretion (ADME) analysis			
		Drug-likeness test			

Multidimensional in silico strategy for identification of natural polyphenols-based SARS-CoV-2 main protease (Mpro) inhibitors to unveil a hope against COVID-19	Adem et al., [33]	Quantum mechanics	SARS-CoV-2 main protease (M^pro^)	Hesperidin, rutin, diosmin, and apiin	None
		Molecular docking			
		Molecular dynamic simulations			

Multi-step in silico discovery of natural drugs against COVID-19 targeting main protease	Elkaeed et al., [34]	Molecular similarity detection using	SARS-CoV-2 main protease	Luteoside C, kahalalide E, and streptovaricin B	None
		Discovery Studio software			
		Molecular fingerprint detection using			
		Discovery Studio software			
		Docking studies using MOE.14 software			
		Toxicity studies using discovery			
		Studio 4.0			
		Molecular dynamics (MD) simulations using the GROningen MAchine			

Natural-like products as potential SARS-CoV-2 Mpro inhibitors: in-silico drug discovery	Ibrahim et al., [35]	Virtual screening of MolPort database	SARS-CoV-2 M^pro^	Four bis [1, 3] dioxolo pyran-5-carboxamide derivatives	None
		Molecular docking			
		Molecular			
		Dynamics (MD) simulations			
		Drug-likeness predictions			

Potent toxic effects of Taroxaz-104 on the replication of SARS-CoV-2 particles	Rabie [37]	Computational molecular docking studies	RNA-dependent RNA polymerase (nCoV-RdRp)	Taroxaz-104	*In vitro* anti-COVID-19 bioactivities of Taroxaz-104
		*In vitro* anti-COVID-19 bioactivities of Taroxaz-104			

Promising terpenes as SARS-CoV-2 spike receptor-binding domain (RBD) attachment inhibitors to the human ACE2 receptor: an integrated computational approach	Muhseen et al., [38]	Structure-based virtual screening	SARS-CoV-2 spike receptor-binding domain (RBD)	NPACT01552, NPACT01557 and NPACT00631	None
		Molecular dynamics (MD) simulation			

Rational design of potent anti-COVID-19 main protease drugs: an extensive multi-spectrum in silico approach	Ahmad et al., [36]	Structure-based virtual screening (SBVS) of ASINEX antiviral library	SARS-CoV-2 M^Pro^	SCHEMBL 12616233, SCHEMBL 18616095, and SCHEMBL 20148701	None
		Drug-likeness and lead likeness annotations			
		Pharmacokinetics analysis			
		Molecular dynamics (MD) simulations			

Rutin and flavone analogs as prospective SARS-CoV-2 main protease inhibitors: in silico drug discovery study	Ibrahim et al., [39]	Virtual screening	SARS-CoV-2 M^pro^	PubChem-129-716-607 and pubChem-885-071-27	None
		Molecular docking			
		Molecular dynamics simulations			
		Drug-likeness evaluation			

Screening, molecular simulation and in silico kinetics of virtually designed Covid-19 main protease inhibitors	Aleissa et al., [40]	Virtual screening	SARS-CoV-2 M^pro^	HIT-1 and HIT-2	None
		Molecular docking			
		Molecular dynamics (MD) simulations			
		ADME calculations			

Structure-based screening of natural product libraries in search of potential antiviral drug leads as first-line treatment for COVID-19 infection	Rao and Shetty [41]	Virtual screening	SARS-CoV NSP12 polymerase	12,28-Oxa-8-hydroxy-manzamine A	None
		Pharmacokinetic and pharmacodynamics properties analysis			
		Molecular docking			
		Molecular dynamic simulations			

Targeting SARS-CoV-2 RNA-dependent RNA polymerase: an in silico drug repurposing for COVID-19 [version 1; peer review: 2 approved]	Baby et al., [42]	Molecular docking	SARS-CoV-2 RNA-dependent RNA polymerase	Pitavastatin, ridogrel, and rosoxacin	None
		Molecular dynamics simulation			

Targeting SARS-CoV-2 spike protein of COVID-19 with naturally occurring phytochemicals: an in silico study for drug development	Pandey et al., [43]	Molecular docking	SARS-CoV-2 spike protein	Fisetin, quercetin, and kaempferol	None
		Molecular dynamics (MD) simulation			
		ADME analysis			

The potential effects of clinical antidiabetic agents on SARS-CoV-2	Qu et al., [44]	Molecular dynamics simulation	SARS-CoV-2 M^pro^	Repaglinide, canagliflozin, glipizide, gliquidone, glimepiride, and linagliptin	*In vitro* study
		Molecular docking study			
		*In vitro* study			

Virtual screening-driven drug discovery of SARS-CoV2 enzyme inhibitors targeting viral attachment, replication, post-translational modification and host immunity evasion infection mechanisms	Quimque et al., [45]	Molecular docking	SARS-CoV2 PLpro	Three fumiquinazoline alkaloids scedapin C, quinadoline B, and norquinadoline A	None
		Molecular dynamics simulation	Chymotrypsin-like protease (3CLpro)	The polyketide iso-chaetochromin	
		Drug-likeness, ADME, and toxicity prediction	SARS-CoV-2 RdRp	The terpenoid 11a-de hydroxy isoterreulactone A	
			SARS-CoV-2 nsp15		
			SARS-CoV-2 S protein (spikes)		

## Data Availability

The data supporting the findings of this study are available upon request from the corresponding author.
